# Predicting intradialytic hypotension in critically ill patients undergoing intermittent hemodialysis: a prospective observational study

**DOI:** 10.1186/s40635-024-00676-x

**Published:** 2024-09-27

**Authors:** Rogério da Hora Passos, Fernanda Oliveira Coelho, Juliana Ribeiro Caldas, Erica Batista dosde Santos GalvãoMelo, Augusto Manoel de Carvalho Farias, Octávio Henrique Coelho Messeder, Etienne Macedo

**Affiliations:** 1 Departamento de Pacientes Graves, Hospital Israelita Albert Einstein, Av Albert Einstein, 627/701, Morumbi, São Paulo, SP Brazil; 2 Davita Tratamento Renal, Rio de Janeiro, Brazil; 3 Escola Bahiana de Medicina e Saúde Pública, Salvador, Brazil; 4 Hospital São Rafael, Salvador, Brazil; 5 Hospital Português, Salvador, Brazil; 6 Nephrology Division, University of California, San Diego, USA

**Keywords:** Acute kidney injury, Dynamic arterial elastance, Hemodynamic monitoring, Intradialytic hypotension, Kidney replacement therapy, Passive leg raising test, Vasomotor tone

## Abstract

**Background:**

Hypotension during dialysis arises from vasomotor tone alterations and hypovolemia, with disrupted counterregulatory mechanisms in acute kidney injury (AKI) patients. This study investigated the predictive value of preload dependency, assessed by the passive leg raising (PLR) test, and arterial tone, measured by dynamic elastance (Eadyn), for intradialytic hypotension (IDH).

**Methods:**

In this prospective observational study conducted in a tertiary hospital ICU, hemodynamic parameters were collected from critically ill AKI patients undergoing intermittent hemodialysis using the FloTrac/Vigileo system. Baseline measurements were recorded before KRT initiation, including the PLR test and Eadyn calculation. IDH was defined as mean arterial pressure (MAP) < 65 mmHg during dialysis. Logistic regression was used to identify predictors of IDH, and Kaplan–Meier analysis assessed 90-day survival.

**Results:**

Of 187 patients, 27.3% experienced IDH. Preload dependency, identified by positive PLR test, was significantly associated with IDH (OR 8.54, 95% CI 5.25–27.74), while baseline Eadyn was not predictive of IDH in this cohort. Other significant predictors of IDH included norepinephrine use (OR 16.35, 95% CI 3.87–68.98) and lower baseline MAP (OR 0.96, 95% CI 0.94–1.00). IDH and a positive PLR test were associated with lower 90-day survival (*p* < 0.001).

**Conclusions:**

The PLR test is a valuable tool for predicting IDH in critically ill AKI patients undergoing KRT, while baseline Eadyn did not demonstrate predictive value in this setting. Continuous hemodynamic monitoring, including assessment of preload dependency, may optimize patient management and potentially improve outcomes. Further research is warranted to validate these findings and develop targeted interventions to prevent IDH.

## Background

Several mechanisms are involved in the pathophysiological basis that leads to hypotension during the dialysis procedure, such as vasomotor tone changes, and hypovolemia. In addition, counterregulatory mechanisms to hypotension may be deregulated in patients with acute kidney injury (AKI) dialysis [1, 2].

The assessment of preload dependence can help define the fluid removal rate with a potential impact on decreasing the frequency of hypotension, especially in patients with acute circulatory failure [3]. Monnet et al. described using passive leg raising (PLR) to discriminate patients with hypotension and decreased cardiac output (CO) after starting dialysis [4].

Changes in systemic vascular resistance (SVR) are frequent in patients with AKI, leading to hemodynamic instability at the precapillary and venous levels [5]. However, relying solely on systemic vascular resistance as an indicator of the arterial system is not sufficient for comprehensive evaluation [6]. Dynamic arterial elastance (Eadyn) has been suggested as a functional assessment of arterial load. It can be used as a predictor of change in arterial pressure after a volume challenge in preload-dependent patients or after changing the noradrenaline infusion rate [7, 8].

Thus, we hypothesized that variables reflecting preload dependency and arterial tone might be associated with arterial pressure variation after initiating kidney replacement therapy (KRT). This study aims to evaluate the clinical utility of using the PLR test and the measurement of Eadyn performed before the start of intermittent hemodialysis as predictors of hemodynamic instability.

## Methods

This was a prospective observational single-center study performed between January 1, 2015, and April 30, 2018, in a 30-bed medical intensive care unit of a tertiary hospital in Salvador, Brazil. The study was approved by the Ethics Committee of Ethical Committee from Centro de Estudos Egaz Muniz (CAAE: 89428318.000005029). Written informed consent was waived for this observational and noninterventional study.

Critically ill patients were included in the study if they fulfilled the following criteria: (i) age > 18 years, (ii) AKI defined by KDIGO 3, (iii) the first intermittent hemodialysis session in the intensive care unit for each patient during the same hospital stay, as decided and prescribed by the nephrologist on duty, (iv) and an indwelling radial artery catheter connected to the FloTrac/Vigileo hemodynamic monitoring system (Edwards LifeSciences, Irvine, CA). Patients were excluded from the study if they had right ventricle dysfunction, significant valvular disease, lung hyperinflation, increased abdominal pressure, pronounced respiratory motion of the inferior vena cava, were using compression stockings, or had no ultrafiltration prescription. Additionally, patients with significant fluctuations in vascular tone or compliance were also excluded, as these conditions could affect the accuracy of cardiac output measurements obtained with the Vigileo/FlowTrac system.

### Intermittent hemodialysis sessions

The nephrology team in charge of patient care was responsible for the indication, timing of initiation of dialysis and prescription. Intermittent hemodialysis sessions were performed based on standard clinical guidelines [9] including AKI with hemodynamic stability, ongoing hypercatabolism, hyperkalemia, severe acidosis, presumed volume accumulation, and respiratory distress. In our center, intermittent hemodialysis is performed in patients without vasopressors or in a low dose of vasopressors (norepinephrine dose ≤ 0.3 mcg kg − 1 min^ − 1^) for at least 6 h before initiation of dialysis, with MAP ≥ 65 mmHg. Clinical, laboratory, and hemodynamic variables were used for each patient to inform clinical decision-making of the ultrafiltration rate. Intermittent hemodialysis was performed with Fresenius 4008 S dialysis machine and dialysate concentrate solutions with 1.75 mmol/L calcium concentration.

### Study design

At baseline, before connecting the patient to the KRT circuit, hemodynamic measurements were registered, and the PLR test was performed. Intradialytic hypotension (IDH) was defined as the occurrence of a MAP below 65 mmHg during the dialysis session. Hemodialysis duration (min), blood flow rate, dialysate flow rate (300 or 500 ml/min), ultrafiltration (UF) volume, dialysate sodium concentration (mEq/L), and dialysate temperature (°C) prescribed were recorded. UF rate prescribed was calculated as ultrafiltration volume per duration in hours and normalized to body weight (ml/kg/h) using the ideal body weight.

### Hemodynamic measurements

The bedside monitor connected to the FloTrac pressure transducer recorded the arterial pressure signal. Variables were recorded every 20 s for Vigileo-derived parameters and arterial pressure waveform recordings. The average of six consecutive measurements for mean arterial pressure (MAP), systolic pressure (SAP), diastolic pressure (DAP), CO, and arterial pulse pressure (PP) was used for statistical analysis.

Eadyn was calculated as the pulse pressure variation (PPV) ratio to stroke volume variation (SVV). SVR was calculated using the formula SVR = (MAP—central venous pressure (CVP)) × 80/CO. The ratio of pulse pressure (SAP—DAP) to stroke volume (PP/SV) was also calculated as a rough measure of arterial stiffness. Although this index may underestimate total arterial stiffness compared to other methods, it has been proven useful for estimating and detecting changes in arterial stiffness in clinical settings [7].

PPV was calculated using the difference between SAP and DAP obtained from the bedside monitor. The highest (Pulse Pressure max) and lowest (Pulse Pressure min) differences were identified over three consecutive respiratory cycles. The average values of these three measurements were used to determine arterial pulse pressure variability. This was calculated using the formula: PPV = (Pulse Pressure max—Pulse Pressure min) / [(Pulse Pressure max + Pulse Pressure min)/2] × 100, as previously explained.

The patient's posture was altered from semi-reclined to PLR, with the legs elevated at a 45° angle and the torso kept horizontal. The bed was adjusted without making direct contact with the patient. The maximum cardiac index measurement obtained through pulse contour analysis during the PLR examination was documented. Typically, this measurement is achieved within a minute [10].

### Statistical analysis

Data were tested for normality by visual inspection and using the Kolmogorov–Smirnov test. Continuous variables were expressed as mean or median (interquartile range, IQR) as appropriate and were compared by Mann–Whitney test. The χ2 test compared proportions. Variables were compared between groups of sessions (with hypotension vs. without hypotension). The binary classification (“no intradialytic hypotension” vs. “intradialytic hypotension”) was used as an outcome variable in a way that “no hypotension” and “hypotension” were coded as 0 and 1, respectively. Quantitative and qualitative variables associated with hypotension with a p-value below 0.05 in univariate analysis were selected for a multivariable logistic regression model. Logistic regression with categorical and continuous independent variables was used to build predictive models for hypotension. Statistical significance was assumed at the 5% level. We employed Kaplan–Meier survival analysis to estimate the cumulative survival probability over the 90-day follow-up period. Patients were categorized into two groups: those who experienced intradialytic hypotension events and those who did not. Survival time was measured from the initiation of the study until death or the end of the 90-day follow-up. To assess differences in survival curves between the two groups, log-rank tests were conducted. Statistical analysis was performed using SPSS version 26.0 for Windows (SPSS Inc., Chicago, IL).

## Results

From January 2015 to April 2018, a total of 187 patients with AKI who required intermittent hemodialysis with ultrafiltration were considered eligible for the study, of whom 104 (55.6%) were male. The median age of the group was 67 (57–76) years. The median Charlson score was 10 (8–12), while the median APACHE II score was 16 (13–18). The median SOFA score was 8 (6–10), and the overall mortality rate within 28 days was 17.1%. Mechanical ventilation was used in 23 patients (12.3%), with the highest recorded positive end-expiratory pressure (PEEP) value being 10 cmH₂O. Further patient characteristics are displayed in Table 1. Median dialysis duration was 240 (210–240) min, and 21 (11.2%) patients had a dialysate flow rate of 300 ml/min, with median UF rate of 5.56 (3.96–6.94) ml/kg/h. Dialysis prescription parameters are detailed in Table 2. Dialysis had to be stopped immediately for hemodynamic improvement in 10 (5.3%) patients experiencing hypotension.

**Table 1:** Clinical, biological and hemodynamic characteristics of patients depending on the occurrence or absence of intradialytic hypotension

	Total	Intradialytic hypotension		*P*
		No	Yes	
N	187	136 (72.7%)	51 (27.3%)	
Clinical characteristics				
Sex				0.904
Male, *n* (%)	104 (55.6%)	76 (55.9%)	28 (54.9%)	–
Female, *n* (%)	83 (44.4%)	60 (44.1%)	23 (45.1%)	–
Age (years)	67 (57.0–76.0)	66 (54–76)	68 (62–75)	0.267
Charlson score	10 (8–12)	10 (8–12)	10 (8–12)	0.216
APACHE II	16 (13–18)	15 (13–18)	16 (13–18)	0.255
SOFA	8 (6–10)	8 (6–10)	8 (6–12)	0.113
Sepsis, *n* (%)	90 (48.1%)	57 (41.9%)	33 (64.7%)	< 0.01
Norepinephrine use at baseline, *n* (%)	24 (12.8%)	5 (3.7%)	19 (37.3%)	< 0.001
Mechanical ventilation, *n* (%)	23 (12.3%)	11 (8.1%)	12 (23.5%)	< 0.01
PEEP, cm H₂O	8(6–8)	8(6–8)	8(5–8)	0.608
28-day mortality *n* (%)	32 (17.1%)	16 (11.8%)	16(31.4%)	< 0.01
Biochemical characteristics				
Hemoglobin, g/dL	9.0 (7.9–10.0)	9.0 (7.8–10.0)	9.0 (8–11)	0.473
Bicarbonate, mEq/L	20 (18–23)	20 (18–23)	20 (18–22)	0.566
Sodium, mEq/L	138 (135–141)	138.5 (135–141)	138 (134–141)	0.656
Urea, mg/dL	142 (111–187)	142.5 (109–192)	136 (116–177)	0.839
Lactate, mmol/L	1.4(1.1–1.9)	1.3 (1.02–1.7)	1.6 (1.2–2.2)	0.01
Last 24 h fluid balance, mL	1780 (1540–2340)	1772 (1532.5–2315)	1790 (1570–2360)	0.595
Hemodynamic parameters				
Systolic arterial pressure, mmHg	132 (116–152)	137 (121.3–160)	114 (103–132)	< 0.001
Diastolic arterial pressure, mmHg	70 (61.0–80.0)	70.5 (64–85.8)	64 (58–70)	< 0.001
Mean arterial pressure, mmHg	89.7 (79.0–104.3)	94.2 (84.1–108.6)	80 (75–88.4)	< 0.001
Heart rate, beats per min	89.0 (78.0–96.0)	89.5 (79.5–97.5)	86 (76–96)	0.422
Positive PLR, *n* (%)	58 (31%)	25 (18.4%)	33 (64.7%)	< 0.001
Eadyn	1.04 (0.91–1.06)	1.04 (0.92–1.06)	1.04 (0.88–1.06)	0.366
Pulse pressure, mmHg	62 (48.0–80.0)	67 (50–81)	53 (40–68)	< 0.001
Cardiac index, mL/min/m^2^	3.2 (2.8–3.5)	3.2 (2.8–3.5)	3.2 (2.6–3.4)	0.328

*APACHE* Acute Physiology and Chronic Health Evaluation, *Eadyn* dynamic arterial elastance, *PEEP* positive end-expiratory pressure, *PLR* passive leg raising test, *SOFA* Sequential Organ Failure Acute

**Table 2:** Dialysis prescription of patients depending on the occurrence or absence of intradialytic hypotension

	Total	Intradialytic hypotension		*P*
		No	Yes	
N	187	136 (72.7%)	51 (27.3%)	
Duration (minutes)	240 (210–240)	240 (210–240)	240 (210–240)	0.840
Blood flow rate, mL/min	300 (250–300)	300 (250–300)	250 (200–300)	0.057
Dialysate flow, 300 mL/min (%)	21 (11.2)	9 (6.6)	12 (23.5)	< 0.01
Ultrafiltration volume, mL	1500 (1000–2000)	1500 (1000–2000)	1500 (1000–2000)	0.090
Ultrafiltration rate, ml/h	375 (286–500)	381.5 (289.5–500)	375 (286–500)	0.097
Ultrafiltration rate, ml/kg/h	5.56 (3.96–6.94)	5.66 (4.24–7.31)	4.81 (3.68–6.41)	< 0.05
Dialysate sodium, mEq/L	138 (138–140)	138.5 (135–141)	138 (134–141)	0.537
Dialysate temperature, °C	36 (36–36)	36 (36–36)	36 (36–36)	0.494

### Incidence and predictive factors of intradialytic hypotension

IDH occurred in 51 (27.3%) patients, with variables such as sepsis, the use of norepinephrine, mechanical ventilation, and higher predialysis lactate levels being significantly associated with IDH. Additionally, patients with IDH had a higher 28-day mortality rate than those without IDH (Table 1). Predialysis hemodynamic findings showed that lower systolic, diastolic, mean arterial pressure, and pulse pressure were significant factors associated with IDH. Furthermore, IDH was more prevalent in preload-dependent patients, as stated by the passive raising test. Median Eadyn, heart rate and cardiac index did not significantly differ between groups (Table 1). There was a difference in prescription of reduced dialysate flow (300 ml/min) between patients with and without IDH: 23.5% of patients with IDH vs 6.6% of patients without IDH (p < 0.01). Ultrafiltration rate was not independently associated with hypotension (Table 3). The logistic regression model for IDH showed that the use of norepinephrine, mean arterial pressure and preload dependence detected by the PLR test were significant and independent predictors of IDH (Table 3).

**Table 3:** Multivariate logistic regression analysis for clinical characteristics, biological, and predialytic hemodynamic characteristics in patients with intradialytic hypotension

Variable	Parameter estimated	Standard error	Odds ratio	CI 95%	*P*
Clinical characteristics					
Sepsis	0.721	0.471	2.057	0.82–5.18	0.13
Use of norepinephrine	2.799	0.743	16.436	3.83–70.55	< 0.001
Mechanical ventilation	0.801	0.767	0.449	0.10–2.02	0.30
Predialytic hemodynamic characteristics Mean arterial pressure, mmHg	0.037	0.017	0.963	0.93–0.99	< 0.05
Positive PLR	2.242	0.476	9.409	3.70–23.92	< 0.001
Pulse pressure, mmHg	0.003	0.011	1.003	0.98–1.03	0.82
Predialytic biochemical characteristics Lactate, mmol/L	0.239	0.344	0.788	0.40–1.55	0.49
Dialysis prescription characteristics Dialysate flow, 300 mL/min	1.041	0.676	2.374	0.094–1.328	0.12
Ultrafiltration rate, ml/kg/h	0.164	0.108	0.849	0.687–1.049	0.13
Constant	2.795				

*PLR* passive leg raising test

### Survival analysis

**Fig. 1:**
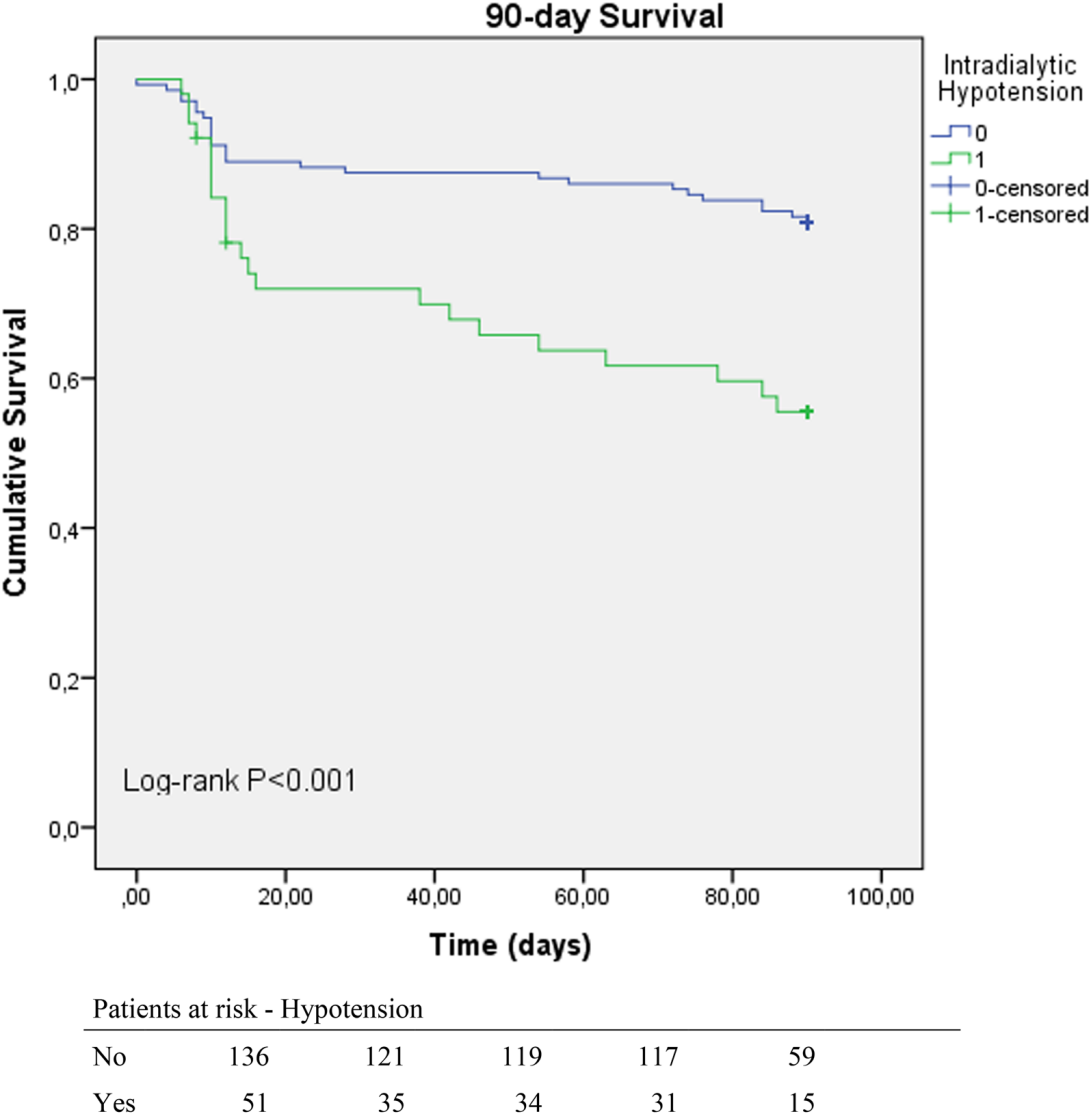
Kaplan–Meier curves showing the survival probability among individuals who encountered intradialytic hypotension compared to those who did not during the 90-day observation period. Patients who experienced episodes of intradialytic hypotension had a notably lower likelihood of survival, with a significant difference (log rank *p* < 0.001)

**Fig. 2:**
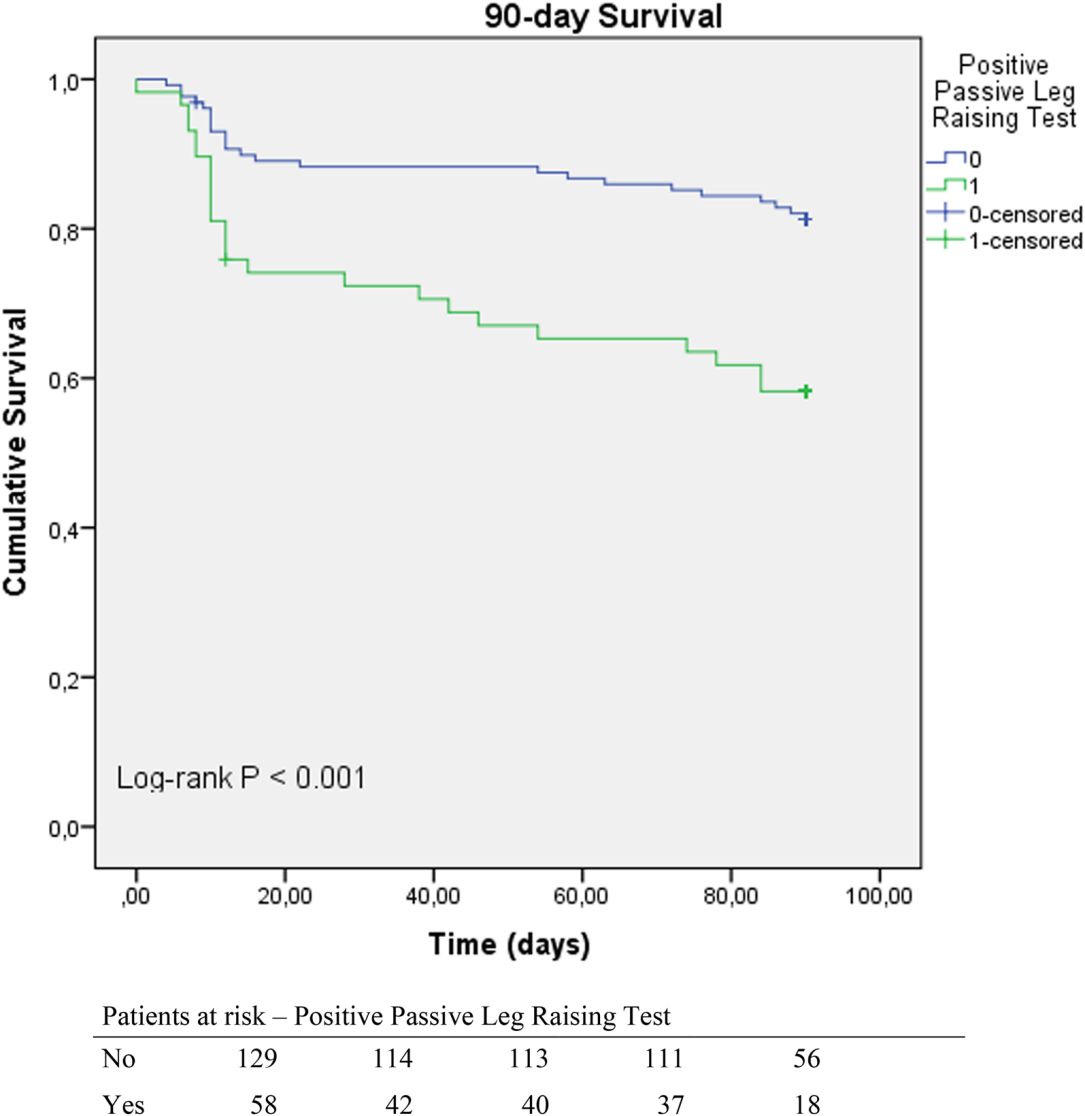
Kaplan–Meier curves showing the survival probability among patients with a positive PLR test compared to those with a negative PLR test. Patients with a positive PLR test had a significantly lower probability of survival, with a significant difference (log rank *p* < 0.001)

The Kaplan–Meier survival curve showed a clear distinction between individuals who encountered intradialytic hypotension and those who did not. During the 90-day observation period, the likelihood of survival was notably lower among patients who experienced episodes of intradialytic hypotension. Likewise, the survival probabilities differed significantly between patients with PLR test and those with a negative PLR test, with the former group having a significantly lower probability of survival. These findings are depicted in Figs. 1 and 2.

## Discussion

In this research, it was observed that patients suffering from intradialytic hypotension demonstrated increased preload dependence as identified by the PLR test. Nevertheless, Eadyn was ineffective in predicting this outcome. Excessive fluid removal during dialysis is a primary contributor to intradialytic hypotension. From a physiological perspective, this problem is likely to manifest when there is preload responsiveness, meaning the heart operates on the steep, initial segment of the cardiac function curve. In such circumstances, a reduction in preload can lead to decreased CO and lower blood pressure [4, 11].

Elevated arterial lactate, high SOFA scores, and reduced cardiac output are linked to an increased risk of preload-dependence-related IDH [12]. Assessing the status of preload dependence or independence during an IDH episode is challenging and necessitates functional hemodynamic monitoring along with continuous measurements of cardiac index [12, 13]. Ongoing hemodynamic assessments are crucial for identifying potential independent risk factors for IDH linked to preload dependence. Extensive research and meta-analyses confirm the reliability of the PLR test for assessing preload responsiveness, demonstrating high sensitivity and specificity (85% and 91%, respectively) with excellent predictive values and likelihood ratios. Its application is now recommended in managing the hemodynamics of critically ill patients [14]. Performing the PLR test before fluid removal can accurately predict intradialytic hypotension, especially due to its high specificity and positive predictive value [4]. Our study found significant associations between IDH and variables such as sepsis, norepinephrine use at baseline, mechanical ventilation, and elevated predialysis lactate levels. Additionally, logistic regression models indicated that the use of norepinephrine and preload dependence identified by the PLR test are independently predictive of IDH.

Vasoplegia and vasodilatory shock are commonly seen in critically ill patients in the ICU, especially those with AKI and multiorgan dysfunction. These patients often experience activation of various intrinsic vasodilatory pathways and a decreased response to vasopressors. Sepsis, in particular, is a frequent condition in critically ill patients and is a major cause of AKI and hemodynamic instability. The underlying mechanisms of impaired vascular tone involve the dysregulation of several mediators, including nitric oxide, inflammatory cytokines, prostaglandins, and complements. SVR has been utilized to describe the arterial system and provide a basic understanding of arterial load. However, more than relying solely on SVR to represent the entire arterial system is required [6, 15].

Eadyn quantifies the relationship between pulse pressure and stroke volume variation, capturing the dynamic interplay between blood pressure and stroke volume throughout a respiratory cycle. It is utilized to predict arterial pressure responsiveness to fluid challenges in preload-dependent patients. A higher Eadyn score suggests a greater likelihood that an increase in CO will also elevate blood pressure. Conversely, a low Eadyn score means arterial blood pressure may not rise even if CO does. Consequently, Eadyn can ascertain whether hypotensive patients might benefit from fluid administration or require vasopressors to boost arterial pressure, aiding in weaning off vasopressors [16, 17]. Our study's predialysis hemodynamic data revealed that lower systolic, diastolic, and mean arterial pressures, along with lower pulse pressure, were significantly associated with IDH. However, in our logistic regression analysis, pulse pressure did not emerge as an independent predictor of IDH. Our findings also indicate that Eadyn was ineffective in predicting a drop in arterial pressure in hemodialysis patients. To our knowledge, this is the first study to investigate Eadyn's utility in predicting hypotension due to fluid removal as opposed to infusion. Several important questions need to be addressed in the context of hypotension in KRT. If necessary, the therapeutic measures to correct it should focus on the underlying causes rather than just addressing the symptoms. Firstly, will CO decrease with fluid removal? If so, will this decrease in CO lead to hypotension? If the answer to both questions is yes, then the first option should be to stop fluid removal. We found that passive leg raising was able to discriminate patients who had hypotension, however, the ultrafiltration rate and Eadyn were not independently correlated with it. Therefore, other mechanisms as ventriculo-arterial coupling, myocardial stunning, rapid osmotic shift may be correlated with intradialytic hypotension, and reducing the ultrafiltration rate alone would not be sufficient to avoid hypotension [18, 19]. It has been reported that maintenance hemodialysis patients may undergo temporary cardiac dysfunction during dialysis, which is indicated by abnormalities in regional wall motion. This dysfunction seems to be linked to reduced myocardial perfusion in the absence of atherosclerotic coronary artery disease. It is worth noting that this phenomenon is separate from the removal of excess fluid [20].

Our findings align with previous research on the prognosis of patients with acute kidney injury (AKI) who require kidney replacement therapy (KRT) and the incidence of intradialytic hypotension (IDH) [22–24]. However, it is important to recognize the variability in IDH definitions across studies. Some research uses absolute thresholds for systolic blood pressure (SBP) or mean arterial pressure (MAP), while others consider relative drops in blood pressure, symptoms, or the need for interventions [25]. In our study, we found that a nadir MAP of less than 65 mmHg was associated with reduced survival. This supports the notion that nadir MAP thresholds are more reliable indicators of IDH-related mortality risk compared to definitions based solely on blood pressure drops or symptoms, even in stable chronic patient cohorts [26]. Additionally, patients with a nadir MAP < 65 mmHg experienced a significantly greater drop in MAP from their initial measurement to the lowest value reached (delta MAP) compared to patients without hypotension.

Continuous kidney replacement therapy (CKRT) is generally preferred for patients with severe hemodynamic instability, while intermittent hemodialysis (IHD) is often used for patients transitioning toward recovery, balancing clinical needs with resource availability. The choice to use IHD is influenced by individual patient needs, clinical judgment, and resource constraints. At our center, patients are typically transitioned to IHD during the de-escalation phase of therapy. This transition is guided by the physician’s discretion and is accompanied by careful monitoring to manage potential risks.

Our study found that patients experiencing episodes of intradialytic hypotension had significantly lower survival rates. Additionally, survival probabilities varied notably between patients with positive and negative passive leg raise (PLR) tests. Recognizing preload dependence before initiating dialysis could lead to more judicious volume removal strategies, potentially favoring CKRT in such cases.

Although ultrafiltration (UF) rate is known to predispose patients to intradialytic hypotension (IDH) [18], our data showed that only the UF rate normalized to body weight differed between hypotensive and non-hypotensive groups, and this difference was not significant in multivariate analysis. It is worth noting that the median fluid balance of 1780 ml over the preceding 24 h likely influenced the nephrologist’s decision regarding UF volume, contributing to lower UF rates. This underscores the importance of assessing not only the UF rate, but also fluid responsiveness, as it significantly impacts the occurrence of intradialytic hypotension and may ultimately influence outcomes such as mortality.

Our study has several limitations that should be considered. Firstly, it was conducted at a single center, suggesting that replication of our findings in multicenter randomized controlled trials would be beneficial for validating our results and hypotheses. Additionally, we did not record data on interventions such as the initiation or escalation of fluids, vasopressors, or medications like hydrocortisone, which could influence the frequency of hypotension. While this omission reflects real-world clinical practice, where such interventions are often adjusted dynamically, capturing this data could have enabled a more comprehensive analysis.

Another limitation is that we measured the end-diastolic area (Eadyn) only at a single point just before dialysis, which may not fully capture potential decreases in blood pressure and cardiac function that occur after fluid removal. Furthermore, Eadyn has not been validated for assessing fluid removal, but rather for evaluating the impact of fluid challenge on blood pressure. Our study also only included a single assessment of preload dependence using the passive leg raise (PLR) test at the start of dialysis. Continuous assessment of preload dependence would provide more detailed information on the occurrence of intradialytic hypotension.

Moreover, while we recorded dialysate temperature, we did not measure patient core temperature before and after intermittent hemodialysis (IHD). The dialysate temperature was consistently set at a median of 36 °C in both groups, reflecting standard clinical practices aimed at balancing patient comfort with hemodynamic stability [21].

Despite these limitations, our study underscores the need for further research in this area and highlights potential directions for future inquiry [22–24].

## Conclusions

In critically ill patients, preload dependence, as assessed by a positive PLR test before starting KRT, predicts hemodynamic intolerance. Eadyn was poorly able to predict it. Further studies should be considered to validate these results and personalize the techniques of dialysis according to the risk of intradialytic hypotension.

## Data Availability

The datasets generated during and/or analyzed during the current study are available from the corresponding author on reasonable request.

## Abbreviations

AKI: Acute kidney injury
CO: Cardiac output
DAP: Diastolic pressure
Eadyn: Dynamic arterial elastance
IDH: Intradialytic hypotension
KRT: Kidney replacement therapy
MAP: Mean arterial pressure
PLR: Passive leg raising
PP: Arterial pulse pressure
PPV: Pulse pressure variation
SAP: Systolic pressure
SVR: Systemic vascular resistance
UF: Ultrafiltration
